# Potential research priorities for understanding and treating severe paranoia (persecutory delusions): a priority-setting partnership between patients, carers, mental health staff, and researchers

**DOI:** 10.1136/bmjment-2024-301224

**Published:** 2024-12-03

**Authors:** David Ariel Sher, Thomas Kabir, Maurice Arbuthnott, Suzie Nettleton, Pauline Dixon, Joanna May, Alvaro Barrera, Poppy Brown, Sarah Reeve, Louise Isham, Felicity Waite, Daniel Freeman

**Affiliations:** 1 Department of Experimental Psychology, University of Oxford, Oxford, UK; 2 Department of Psychiatry, University of Oxford, Oxford, UK; 3 Advisory Group, Oxford, UK; 4 Berkshire Healthcare NHS Foundation Trust, Bracknell, Bracknell Forest, UK; 5 Oxford Health NHS Foundation Trust, Oxford, UK; 6 Oxford Institute of Clinical Psychology Training and Research, University of Oxford, Oxford, UK; 7 Norwich Medical School, University of East Anglia, Norwich, UK; 8 Cambridgeshire and Peterborough NHS Foundation Trust, Cambridge, Cambridgeshire, UK

**Keywords:** Schizophrenia & psychotic disorders, PSYCHIATRY

## Abstract

**Background:**

A persecutory delusion (severe paranoia) occurs when a person believes that others are trying to harm them when they are not. It is often a central difficulty for patients diagnosed with schizophrenia.

**Objective:**

The objective is to identify potentially important research questions about severe paranoia.

**Methods:**

A priority-setting partnership exercise was conducted involving people with lived experience, carers, mental health staff, and researchers. An initial survey identified research questions, and a second survey prioritised a refined list of questions. There was a project steering group.

**Findings:**

1480 responses were gathered from 146 people (56 people with lived experience, 23 family members, 78 mental health staff, and 21 researchers). Following refinement, 201 questions were rated by the steering group for priority to enter the second survey. 38 questions were rated in the second survey by 157 people (69 people with lived experience, 33 family members, 59 mental health staff, and 27 researchers). 15 questions were identified as research priorities, each endorsed to a largely similar extent across stakeholder groups. These covered a wide range of topics, including how to support family and carers, understanding the causes of paranoia, managing paranoid thoughts day-to-day, improving access to services, and developing psychological and pharmacological approaches.

**Conclusions:**

There was a good deal of consensus in key questions—covering many aspects of understanding, treatment, and support—to be answered about severe paranoia. Most questions were considered largely equally important.

**Clinical implications:**

Numerous questions were identified that, if addressed, might improve clinical provision for persecutory delusions.

**WHAT IS ALREADY KNOWN ON THIS TOPIC**

- There have been no research priority-setting studies specifically for severe paranoia. 10 top priorities for schizophrenia research have been identified, but these did not concern specific psychotic experiences, and it is plausible that persecutory delusions could present a number of particular challenges for patients, carers, and staff that require research to address.

**WHAT THIS STUDY ADDS**

- This study has led to the identification of a wide range of important research questions about severe paranoia, including ‘How can families and carers be better equipped and supported to manage severe paranoia in loved ones?’, ‘How do people with severe paranoia manage paranoid thoughts on a day-to-day basis?’ and ‘What are the root causes of severe paranoia?’

**HOW THIS STUDY MIGHT AFFECT RESEARCH, PRACTICE OR POLICY**

- The study has identified many key questions that could be addressed in research. They can be a helpful resource for researchers, and if addressed, the questions will likely improve service provision for patients experiencing clinical levels of paranoia.

## Background

 Severe paranoia comprises a strongly held belief that other people (or organisations or other entities) are deliberately trying to cause you harm when they are not. There is a spectrum of severity of paranoia, consisting of a hierarchy of interpersonal sensitivities, mistrust, ideas of reference, and ideas of persecution.^1 2^ Our focus is on clinical levels of paranoia. The severest form is persecutory delusion, a key difficulty for people diagnosed with conditions such as schizophrenia. Severe paranoia causes patients considerable distress.^3^ Suicidal ideation^4^ and psychiatric hospital admission^5^ are commonplace, and there can be considerable negative impact on family and relationships.^6 7^ Its occurrence can cut across many different mental health diagnoses.^8 9^ Paranoia is an area of increasing research on its causes and treatment. We set out to learn from key stakeholders—patients, carers, mental health staff, and researchers—what may be the key priorities for research on severe paranoia.

When researchers and clinicians prioritise research, they may believe their research priorities are widely held, and that they mirror the research priorities of patients. However, the research priorities of researchers and clinicians can be mismatched to a variable degree from patient priorities.^10 11^ Involving patients in formulating research priorities can produce research that is more relevant and tailored to patient needs.^12 13^ Priority-setting partnerships (PSPs) aim to create a list of research priorities jointly agreed on by people from several different backgrounds (e.g., carers, clinicians, and patients). The James Lind Alliance (JLA) has pioneered this approach in the UK and produces lists of the ‘top 10’ research priorities in several areas.^14^ There are discussions of how such exercises should best be conducted.^15^

A PSP has produced a list of the top 10 schizophrenia research priorities.^14 16^ The study did not aim to cover specific symptoms associated with schizophrenia, such as persecutory delusions. There was no mention of specific psychotic experiences in the schizophrenia research priorities list, presumably due to the broader focus of the project. Schizophrenia is a diagnosis that covers a wide range of independent psychotic experiences, including paranoia, grandiosity, hallucinations, thought disorder, and anhedonia.^17 18^ Each will have different causes and, therefore, different treatment needs. We think there may be specific challenges associated with individual psychotic experiences, such as severe paranoia, that make a research priority-setting exercise of value. For example, the mistrust inherent in paranoia may affect engagement with services and treatments, create tensions with families and carers, and increase the likelihood of not participating in research that does not reflect their priorities. And although there have been considerable improvements in treatment,^19^ there are still many patients who do not recover with existing treatment options. Greater research on this topic is needed.

## Objective

The aim of this study was to generate for the first time a list of research questions from patients, family members, mental health staff, and researchers on what they consider needs addressing in research on clinical instances of paranoia. This could serve as an aid to improve research on the clinical issue. Our hope was to generate a large set of questions rather than to restrict research with a narrow set of priorities unnecessarily.

## Methods

The methodology drew on the processes of the JLA PSP, but these were modified and not identical.^20^ Our priority-setting process can broadly be conceptualised as consisting of three phases: (1) gathering research questions, (2) refining the research questions and, finally, (3) identifying a list of key questions. In the first phase, a survey gathered potential questions for research on paranoia. Data collection took place through an electronic survey. The survey was accessed via a weblink that was circulated. A PDF version of the survey was available via the survey introductory webpage and could be downloaded and printed by survey responders. On the front page of the survey, severe paranoia was described as: “Paranoia is a form of mistrust. We’re interested in severe paranoia, which is excessive mistrust. The severest form of paranoia is persecutory delusions, which refers to the exaggerated belief that others want to harm the individual. Such beliefs could include the strong conviction that others want to physically hurt the person, or try to hurt them socially.” Potential questions suggested by respondents were tabulated, duplicates were removed, and suggested questions were reformulated into research questions. Questions that were ambiguous or had been previously addressed in research studies were removed. With supervisory guidance, the list of questions was further reduced and the list of refined questions was then entered into a second survey, which responders could access in the same way as the first survey. A PDF version of the second survey was available to download via the survey webpage. Participants selected their top 10 questions in the second survey, and a list of the most-endorsed research priorities for paranoia was then produced.

Oversight of the study was provided by a priority-setting steering group, which comprised three people who self-identify as having lived experience of severe paranoia and attend or had attended mental health services, one family member, two research clinical psychologists, one psychiatrist, one manager of an early intervention in psychosis service, and one DPhil student (DAS). The study documents and surveys were designed following review and editing by people with lived experience of severe paranoia. The PSP was called ‘Setting Priorities for Paranoia Research—Oxford’.

### Participants

A cascade sampling strategy,^21^ via email and research group networks, was used for recruitment across several stakeholder groups, including adults who self-identify as having lived experience of severe paranoia and attend or had attended mental health services, people who self-identify as having lived experience of severe paranoia and do not attend mental health services, family members, National Health Service (NHS) (or other mental health service) mental health professionals, and researchers. Survey responders were able to select more than one identity category. Organisations that were sent the survey included 21 NHS mental health trusts, 5 recovery colleges and 5 mental health charities. One of these charities, the European Federation of Associations of Families of People with Mental Illness (EUFAMI), is a pan-European network organisation representing family members of people affected by mental health, with 38 member organisations across 26 countries. EUFAMI’s European partners were also asked to promote the study. The study was promoted by public and patient involvement colleagues, two early intervention teams, the Department of Psychiatry at the University of Oxford, and five other university research groups focussing on mental health. A study-specific Twitter account was created to develop awareness and engagement with the research.

### Procedure

#### Initial survey

The steering group agreed the content of the first survey to elicit research questions from participants. There were seven main prompts for participants (see below). The content and ordering of these prompts was discussed in a steering group meeting after a draft was circulated by email. For example, a question on managing and living with paranoia was included (‘What research questions on managing and living with severe paranoia do you think should be answered?’) after a steering member with lived experience indicated that this reflected an important area to ask about.

The initial survey covered seven main questions:

1. What research questions on the *treatment* of severe paranoia do you think should be answered?

(Treatment may include medication, talking therapies, and other therapies, etc.)

1. What research questions on the *impact* of severe paranoia do you think should be answered?

(Impact might include physical and emotional impact, impact of paranoia in the workplace, and/or impact on family, relationships, friends, relaxation, etc.)

1. What research questions on *recovery* from severe paranoia do you think should be answered?

(By recovery we mean paranoia having little or no impact on one’s life.)

1. What research questions on *how people view* severe paranoia do you think should be answered?

(This can include stigma, stereotypes or the way family, professionals, psychologists, friends, the media, and the general public talk about or view severe paranoia.)

1. What research questions on *managing and living with* severe paranoia do you think should be answered?
2. What research questions on *NHS (or other mental health service) services* for people with severe paranoia do you think should be answered?

(This can include what would be helpful for service providers to implement to be able to better relate, interact, and communicate with people with severe paranoia.)

1. Are there any other research questions on severe paranoia that you think are important to address?

Participants could enter their suggested questions in an open text box placed beneath each question. Participants were asked their role and could select more than one option (e.g., indicate that they were both someone with lived experience and also a researcher). Participants were also asked their gender, age, ethnicity, and where they live. These questions were optional, and participants could select a ‘prefer not to say’ option.

### Refining the question list

Steering members agreed that decisions on reducing the list of questions would be made by DAS under the supervision of DF and FW, that a log of this would be kept, and that all steering members would be able to access this log. Following tabulation, removal of duplicate questions and reformulating questions into research questions by DAS, the number of suggested questions (‘raw submissions’) was reduced by PB and SR in consultation with DAS. Suggestions were removed if they were not research questions (e.g., ‘Taking therapy’), if the suggestions were already clearly addressed in the research literature (e.g., ‘Is medication effective at all in treating paranoia?’), or if they were ambiguous (e.g., ‘How did it manifest itself?’). Questions were also deemed ambiguous and removed if there could be more than one interpretation of the question’s meaning (e.g., ‘Why do you think that you suffer from paranoia?’). Determination of whether questions had been previously addressed was undertaken by DAS, with the support of four of the study authors where applicable. The four authors were: DF, FW, SR, and PB. Uncertainties were screened against entries in online academic search engines. Discrepancies between PB and SR concerning reduction of the question list were resolved in consultation with DAS. 285 (19%) questions were jointly rated by PB and SR. They had a Cohen’s kappa of 0.67 (i.e., substantial agreement). In consultation with DF, TK, and FW, DAS reduced this number further to create a manageable list to present to steering group members. Further, very similar duplicate questions, questions that had already been addressed, and questions that were not research questions and could not be reformulated due to ambiguity were identified and removed. A common reason for excluding a submission was that it was an audit question rather than a research question. For example, the submission ‘What kind of support was offered or you were signposted to, specifically concerning your paranoid ideation?’ appeared to be an audit question. To ensure transparency, a log of this process and all excluded questions was kept and made available to steering members.

A Qualtrics survey (https://www.qualtrics.com) for the steering group was then created to assist with deciding how to reduce the question list further. All steering group members completed the survey. The survey was presented in simplified multiple choice format to enhance accessibility, and steering group members indicated which questions they thought were strong and should be included in the priority-setting list.

### Second survey: identifying priorities

Participants in the first survey were invited to leave their email addresses to be contacted for participation in the second survey. Responders could choose to not provide email details. 103 of 146 responders to the first survey left email details to enable participation in the second survey. However, recruitment for the second survey extended beyond responders to the first survey, as clinicians could contact different patients on their caseloads and different people may have seen the second survey advertised on social media accounts and organisational websites. 52 email addresses provided by responders in the second survey matched email addresses provided in the initial survey. There were 64 new email addresses in the second survey that did not match those provided in the initial survey. Participants were asked in the second survey to select the top 10 questions they thought should be prioritised. The presentation of the questions was randomised to reduce order bias.^22^

### Confirmation of final list

In the final stage, a meeting was held with the steering group to review the findings of the second survey. It was discussed whether any further consultation with stakeholder group representatives was needed to order priorities, what an optimal cut-off point for the selected questions may be (i.e., the number of priorities to present in the final list), and how the findings may be presented.

## Findings

The first completed initial survey response was received on 18 November 2021, and the final initial survey response was received on 16 June 2022. There were 146 survey responders (see Table 1 below). 1480 suggestions for research questions were received overall.

**Table 1:** Information about the survey respondents

	First survey n (%)	Second survey n (%)
Total participants	146	157
Gender		
Prefer not to say	3 (2%)	1 (1%)
Male	61 (42%)	45 (29%)
Female	81 (55%)	105 (67%)
Other	1 (1%)	1 (1%)
Role		
A person with lived experience of severe paranoia who attended/attends mental health services	49 (34%)	57 (36%)
A person with lived experience of severe paranoia who does not attend mental health services	7 (5%)	12 (8%)
A family member of someone with lived experience of severe paranoia	23 (16%)	33 (21%)
An NHS (or other mental health service) mental health professional	78 (53%)	59 (38%)
A researcher	21 (14%)	27 (17%)
Other; please specify:	8 (5%)	4 (3%)
Ethnicity		
Prefer not to say	6 (4%)	2 (1%)
White—English/Welsh/Scottish/Northern Irish/British	101 (69%)	103 (66%)
White—Irish	2 (1%)	4 (3%)
White—Gypsy or Irish Traveller	1 (1%)	0 (0%)
Any other White background	14 (10%)	18 (11%)
Mixed/multiple ethnic groups—White and Black Caribbean	0 (0%)	0 (0%)
Mixed/multiple ethnic groups—White and Black African	1 (1%)	0 (0%)
Mixed/multiple ethnic groups—White and Asian	3 (2%)	2 (1%)
Any other mixed/multiple ethnic group background	3 (2%)	2 (1%)
Asian/Asian British—Indian	2 (1%)	2 (1%)
Asian/Asian British—Pakistani	0 (0%)	1 (1%)
Asian/Asian British—Bangladeshi	1 (1%)	2 (1%)
Asian/Asian British—Chinese	0 (0%)	0 (0%)
Any other Asian background	0 (0%)	1 (1%)
Black/African/Caribbean/Black British—Black/African	2 (1%)	4 (3%)
Black/African/Caribbean/Black British—Black/Caribbean	3 (2%)	6 (4%)
Any other Black/African/Caribbean background	2 (1%)	0 (0%)
Arab	1 (1%)	0 (0%)
Other ethnic group, please specify:	4 (3%) Japanese—1 (1%) Jewish—2 (1%) White Jewish—1 (1%)	4 (3%) Jewish—2 (1%) White Western European—1 (1%) Blank—1 (1%)
Age (years)		
Prefer not to say	7 (5%)	2 (1%)
16–24	7 (5%)	2 (1%)
25–34	35 (24%)	23 (15%)
35–44	25 (17%)	27 (17%)
45–54	29 (20%)	44 (28%)
55–64	26 (18%)	28 (18%)
≥65	14 (10%)	25 (16%)
Residence		
Prefer not to say	8 (5%)	4 (3%)
England—East	12 (8%)	10 (6%)
England—East Midlands	5 (3%)	5 (3%)
England—Greater London	12 (8%)	23 (15%)
England—North East	4 (3%)	2 (1%)
England—North West	9 (6%)	12 (8%)
England—South East	62 (42%)	45 (29%)
England—South West	8 (5%)	7 (4%)
England—West Midlands	4 (3%)	3 (2%)
England—Yorkshire	0 (0%)	6 (4%)
Northern Ireland	0 (0%)	0 (0%)
Scotland	2 (1%)	2 (1%)
Wales	2 (1%)	1 (1%)
Other country, please specify:	18 (12%) Malta—1 (1%) Netherlands—1 (1%) Sweden—5 (3%) UK—8 (5%) USA—3 (2%)	31 (20%) Germany—1 (1%) Ireland—1 (1%) Malta—1 (1%) Netherlands—2 (1%) Portugal—1 (1%) Sweden—3 (2%) UK—20 (13%) USA—2 (1%)

NHS, National Health Service.

The number of suggested questions (‘raw submissions’) was reduced from 1480 raw submissions to 476. The number of raw submissions was then reduced further from 476 to 201. This reduction was undertaken by the first author in consultation with DF and FW, in the context of doctoral supervision meetings.

All nine steering group members completed a Qualtrics survey to determine which of the 201 questions would be entered into the second survey. 38 questions were rated as *‘Strong—should be included’* by a majority of steering group members (see online supplemental material 1).

The second priority-setting survey was released on 3 July 2023 and participants selected the top 10 questions they thought should be prioritised based on their experiences. 157 people completed the survey (see Table 1). The first completed second survey response was submitted on 15 August 2023, and the last survey response on the 2 October 2023.

A score for each question was produced by totalling the number of times that it was in the top 10 questions selected by participants (which could therefore vary from 0 to 157). Questions were then ranked by these scores. The steering committee reviewed the results of the survey during a steering meeting held on 17 November 2023. It was collaboratively agreed that the simple rankings provided a good method for prioritising questions, that the first 15 questions covered a breadth of coverage of issues, that within those questions there were many top priorities shared across stakeholder groups, and that if the report also provided the rankings presented by different stakeholder groups then the aim of the research priority-setting exercise had been achieved. A final workshop was not needed.

Table 2 therefore shows the list of the overall top 15 questions. Table 3 shows the top questions for people with lived experience and family members, and Table 4 shows the top questions for mental health professionals and researchers. Lived experience and family priorities extend to 16 questions, and researcher priorities extend to 19 questions, due to joint 15th question rankings.

**Table 2:** Top 15 questions

Number endorsing	Overall top 15 questions
69 of 157 (44%)	How can families and carers be better equipped and supported to manage severe paranoia in loved ones?
59 of 157 (38%)	How do people with severe paranoia manage paranoid thoughts on a day-to-day basis?
57 of 157 (36%)	What are the root causes of severe paranoia?
56 of 157 (36%)	How can recovery from severe paranoia, especially post-therapy, be best sustained?
55 of 157 (35%)	What characteristics or circumstances before psychosis predict progression from anxiety to severe paranoia?
54 of 157 (34%)	How can severe paranoia be addressed when some of the paranoia may be grounded in true lived experience, such as a violent attack resulting in constant fear of attack?
53 of 157 (34%)	Can peer support and Soteria-informed approaches (approaches which use as little antipsychotic medication as possible and encourage recovery in non-psychiatric settings) work to end severe paranoia?
51 of 157 (32%)	What should come first in treating severe paranoia: pharmacological (medication) intervention or psychological intervention?
50 of 157 (32%)	Are there differences between different ethnic groups in terms of how paranoia presents, is managed, and in recovery?
49 of 157 (31%)	How can access to services for people with severe paranoia be improved?
48 of 157 (31%)	What coping mechanisms are used for severe paranoia?
48 of 157 (31%)	Which medication is most effective for severe paranoia?
47 of 157 (30%)	To what extent does forced hospitalisation and medication add to paranoid ideation?
46 of 157 (29%)	What is the impact on children of having parents who experience severe paranoia?
45 of 157 (29%)	How can GPs and others be best supported to help people with severe paranoia in primary care?

GP, general practitioner.

**Table 3:** People with lived experience and family members’ top priorities for paranoia research

People with lived experience endorsing (n)	Lived experience top questions 69 people	Family members endorsing (n)	Family members’ top questions 33 people
27 of 69 (39%)	What are the root causes of severe paranoia?	22 of 33 (67%)	How can families and carers be better equipped and supported to manage severe paranoia in loved ones?
26 of 69 (38%)	How can severe paranoia be addressed when some of the paranoia may be grounded in true lived experience, such as a violent attack resulting in constant fear of attack?	16 of 33 (48%)	How do people with severe paranoia manage paranoid thoughts on a day-to-day basis?
25 of 69 (36%)	How do people with severe paranoia manage paranoid thoughts on a day-to-day basis?	15 of 33 (45%)	Can peer support and Soteria-informed approaches (approaches which use as little antipsychotic medication as possible and encourage recovery in non-psychiatric settings) work to end severe paranoia?
25 of 69 (36%)	What characteristics or circumstances before psychosis predict progression from anxiety to severe paranoia?	15 of 33 (45%)	How can access to services for people with severe paranoia be improved?
25 of 69 (36%)	What should come first in treating severe paranoia: pharmacological (medication) intervention or psychological intervention?	13 of 33 (39%)	As most NHS (or other mental health service) services work on a model of needing significant engagement from people, what is the best model of engagement to use for people with severe paranoia?
24 of 69 (35%)	What is the impact of severe paranoia on cognitive functioning (e.g., learning, reasoning, remembering, etc)?	12 of 33 (36%)	What coping mechanisms are used for severe paranoia?
23 of 69 (33%)	Could severe paranoia be prevented?	12 of 33 (36%)	To what extent does forced hospitalisation and medication add to paranoid ideation?
22 of 69 (32%)	How can GPs and others be best supported to help people with severe paranoia in primary care?	11 of 33 (33%)	Why does some people’s severe paranoia involve the same paranoid delusions during each experience of an episode? (e.g., why are they always the same/similar beliefs, yet always slightly more advanced each time)
21 of 69 (30%)	Which medication is most effective for severe paranoia?	11 of 33 (33%)	How can recovery from severe paranoia, especially post therapy, be best sustained?
20 of 69 (29%)	How can severe paranoia be managed outside of hospitals when it is life-threatening?	10 of 33 (30%)	Which medication is most effective for severe paranoia?
20 of 69 (29%)	How can families and carers be better equipped and supported to manage severe paranoia in loved ones?	10 of 33 (30%)	What should come first in treating severe paranoia: pharmacological (medication) intervention or psychological intervention?
20 of 69 (29%)	How can recovery from severe paranoia, especially post therapy, be best sustained?	9 of 33 (27%)	What is the impact of caring for someone with severe paranoia?
20 of 69 (29%)	What coping mechanisms are used for severe paranoia?	9 of 33 (27%)	Does hospital help or hinder recovery in individuals with severe paranoia?
20 of 69 (29%)	To what extent does forced hospitalisation and medication add to paranoid ideation?	9 of 33 (27%)	How can the suicide risk of people who have recently recovered from severe paranoia be reduced?
19 of 69 (28%)	Can peer support and Soteria-informed approaches (approaches which use as little antipsychotic medication as possible and encourage recovery in non-psychiatric settings) work to end severe paranoia?	9 of 33 (27%)	How can severe paranoia be managed outside of hospitals when it is life-threatening?
19 of 69 (28%)	What is the impact of severe paranoia on someone’s ability to carry out everyday basic tasks (buying food, cooking, washing, etc)?	9 of 33 (27%)	What characteristics or circumstances before psychosis predict progression from anxiety to severe paranoia?
12 of lived experience top questions match the overall top 15 questions.		10 of family members’ top questions match the overall top 15 questions.	

GP, general practitioner; NHS, National Health Service.

**Table 4:** Mental health professionals’ and researchers’ top priorities for paranoia research

Mental health professionals endorsing (n)	NHS or other mental health service professionals’ top questions	Researchers endorsing (n)	Researchers’ top questions
28 of 59 (47%)	How can families and carers be better equipped and supported to manage severe paranoia in loved ones?	17 of 27 (63%)	How can recovery from severe paranoia, especially post therapy, be best sustained
26 of 59 (44%)	Are there differences between different ethnic groups in terms of how paranoia presents, is managed, and in recovery?	15 of 27 (56%)	How can families and carers be better equipped and supported to manage severe paranoia in loved ones?
25 of 59 (42%)	How can recovery from severe paranoia, especially post-therapy, be best sustained?	13 of 27 (48%)	Are there differences between different ethnic groups in terms of how paranoia presents, is managed, and in recovery?
25 of 59 (42%)	What are the root causes of severe paranoia?	12 of 27 (44%)	Can peer support and Soteria-informed approaches (approaches which use as little antipsychotic medication as possible and encourage recovery in non-psychiatric settings) work to end severe paranoia?
23 of 59 (39%)	For people who have recovered from severe paranoia, what, if anything, about NHS or other mental health services helped them, or did not?	11 of 27 (41%)	What are the root causes of severe paranoia?
23 of 59 (39%)	What characteristics or circumstances before psychosis predict progression from anxiety to severe paranoia?	11 of 27 (41%)	How can severe paranoia be addressed when some of the paranoia may be grounded in true lived experience, such as a violent attack resulting in constant fear of attack?
23 of 59 (39%)	How can severe paranoia be addressed when some of the paranoia may be grounded in true lived experience, such as a violent attack resulting in constant fear of attack?	10 of 27 (37%)	How does illegal drug use (including long-term and short-term drug use) lead to persistent severe paranoia?
22 of 59 (37%)	What is the impact on children of having parents who experience severe paranoia?	10 of 27 (37%)	What can help people with severe paranoia get back to work?
21 of 59 (36%)	How do people with severe paranoia manage paranoid thoughts on a day-to-day basis?	10 of 27 (37%)	What should come first in treating severe paranoia: pharmacological (medication) intervention or psychological intervention?
20 of 59 (34%)	As most NHS (or other mental health service) services work on a model of needing significant engagement from people, what is the best model of engagement to use for people with severe paranoia?	10 of 27 (37%)	What characteristics or circumstances before psychosis predict progression from anxiety to severe paranoia?
19 of 59 (32%)	What does recovery mean for people diagnosed with severe paranoia?	9 of 27 (33%)	For people who have recovered from severe paranoia, what, if anything, about NHS or other mental health services helped them, or did not?
19 of 59 (32%)	How does illegal drug use (including long-term and short-term drug use) lead to persistent severe paranoia?	9 of 27 (33%)	Could severe paranoia be prevented?
19 of 59 (32%)	Can peer support and Soteria-informed approaches (approaches which use as little antipsychotic medication as possible and encourage recovery in non-psychiatric settings) work to end severe paranoia?	9 of 27 (33%)	What coping mechanisms are used for severe paranoia?
18 of 59 (31%)	What coping mechanisms are used for severe paranoia?	9 of 27 (33%)	How can access to services for people with severe paranoia be improved?
18 of 59 (31%)	How can GPs and others be best supported to help people with severe paranoia in primary care?	7 of 27 (26%)	What does recovery mean for people diagnosed with severe paranoia?
		7 of 27 (26%)	What are the types of qualities in NHS or other mental health service professionals that help people with severe paranoia feel more comfortable, better able to trust, and share the difficulties that come about due to severe paranoia?
		7 of 27 (26%)	As most NHS (or other mental health service) services work on a model of needing significant engagement from people, what is the best model of engagement to use for people with severe paranoia?
		7 of 27 (26%)	Which medication is most effective for severe paranoia?
		7 of 27 (26%)	How do people with severe paranoia manage paranoid thoughts on a day-to-day basis?
11 of mental health professionals’ top questions match the overall top 15 questions		12 of researchers’ top questions match the overall top 15 questions	

GP, general practitioner; NHS, National Health Service.

The most-endorsed question overall was ‘How can families and carers be better equipped and supported to manage severe paranoia in loved ones?’, with 69 of 157 (44%) responders to the second survey selecting it as one of their top ten questions. Interestingly, this top overall question was endorsed less among people with lived experience, for whom this question was the 11th most-endorsed question. Notably, levels of overall endorsement across the top 15 priorities did not markedly differ. For example, the tenth most-endorsed question overall was *‘*How can access to services for people with severe paranoia be improved?’, with 49 of 157 (31%) responders to the second survey selecting it as one of their top ten questions. Comparatively, the least-endorsed question of the overall top 15 questions (‘How can general practitioners (GPs) and others be best supported to help people with severe paranoia in primary care?’) was still endorsed by 45 of 157 responders (29%), which illustrates how overall endorsement did not vary greatly across the 15 top priorities. There was a degree of variation across stakeholder groups. For instance, 6 of the 15 top patient priorities were not top priorities of family members, and vice versa. There was also close agreement between the top priorities of mental health service professional and researchers. 13 of the priorities of mental health professionals were also shared by researchers.

## Discussion

A list of 15 questions to be prioritised for paranoia research was identified. No question was endorsed by a majority of respondents. The cut-off for the list of priorities was set at 15, rather than 10 as is common in many such exercises, for three key reasons. First, setting the cut-off at 15 questions ensured that at least 10 research priorities from each stakeholder category featured in the list of research priorities. Second, the tenth most endorsed priority was not an obvious cut-off point, since there was little difference in endorsement rates between questions 10 and 15. The tenth most endorsed priority was endorsed by 49 respondents and the 15th priority was endorsed by 45 respondents. Third, the top 15 priorities covered a wide range of important topics without significant overlap. The topics include the causes of severe paranoia, peer support and Soteria-informed approaches, coping mechanisms, differences between ethnic groups, access to services, sustaining recovery, medication, and the impact on children with parents who experience severe paranoia. We believe this list could be a helpful resource for researchers seeking to make progress in understanding and treating paranoia.

The most-endorsed priority was ‘How can families and carers be better equipped and supported to manage severe paranoia in loved ones?’ It may well be a reflection of how excessive mistrust can negatively impact relationships.^6 7^ It is also plausible that this may reflect insufficient support for families caring for people with psychosis, and disillusionment among carers at not receiving information that could assist in caring for loved ones.^23^ Just as carers may want further information and support in caring for loved ones with paranoia, patients may also want more information on their condition and why they experience it. This may be why the most endorsed research priority by people with lived experience was ‘What are the root causes of severe paranoia?’. The lived experience group in particular also rated highly the question ‘How can severe paranoia be addressed when some of the paranoia may be grounded in true lived experience, such as a violent attack resulting in constant fear of attack?’. The endorsement of this priority (initially submitted by a lived experience participant) highlights the complex relationship between real experiences of negative treatment by other people and inaccurate judgements of hostile intent from others. Mistrust is not necessarily misplaced.^24^ There were differences in emphasis in the most valued research priorities by stakeholder group. This is likely attributable to participants valuing priorities which are more aligned with their everyday experiences. As an illustration of this, ‘How do people with severe paranoia manage paranoid thoughts on a day-to-day basis?’ constituted the third highest priority for lived experience participants, the second highest priority for family members, but the ninth priority for clinicians and the last priority for researchers.

There was little similarity between the schizophrenia PSP^16^ list of questions and questions generated during our process. Questions especially important to people experiencing specific psychotic experiences may be likely to be filtered out in exercises principally focused on schizophrenia because many of the participants may not have experienced a particular psychotic experience. In contrast, all our participants had an interest in paranoia. Broader exercises concerning diagnoses can be valuable, but we believe the current study shows the merit also of examining in detail a single area of patient difficulty.

We consider that researchers should be wary of allocating too much prominence to the order of the list of research priorities for paranoia, for the order of priorities varied somewhat across stakeholder groups and many priorities were rated to a similar extent. Essentially, this was a process of assembling interesting priorities from stakeholders, rather than suggesting certain priorities on the list are more important than others. This approach broadens areas for research rather than limits them. Relatedly, there are many valuable and noteworthy questions in the top 38 research priorities (see online supplemental material 1). These may also constitute important directions for future research. These research priorities should be taken as a starting point. It will be for researchers working together with people with lived experience and others to develop them into research studies.

This study does have several limitations. First, none of the stakeholder groups would have been fully representative since a limited cascade method of recruitment was used.^25 26^ Other biases could also have been introduced. For example, it is conceivable that individuals with high levels of conviction in their persecutory beliefs may have felt too mistrustful to participate in the research. Moreover, targeted sampling to ensure representative levels of participation from distinct stakeholder groups was not undertaken. This shortcoming could have been addressed by employing, for example, a stratified random sampling strategy.^27^ Overall, this is a key limitation, and the list of questions should be used to spur research rather than limit the topics of enquiry. Indeed, it is conceivable that gathering suggestions for research priorities from more participants or within specific sociodemographic groups would reveal additional research priorities to those on our list. The exact ordering of the list would vary by the balance of participants across different stakeholder groups. For example, the top priority overall (‘How can families and carers be better equipped and supported to manage severe paranoia in loved ones?’) was only the eleventh most-endorsed priority by people with lived experience, meaning that it might not have remained the top priority if there was a higher proportion of people with lived experience responding to the survey. Additionally, the priority-setting steering group was relatively small and would not have fully represented those stakeholder groups. Certain groupings were not represented on the steering committee. For example, while clinical psychologists, psychiatrists, and early intervention managers were represented on the steering committee, care co-ordinators were not.

Moreover, this priority-setting study featured multiple levels of filtering of questions. We documented this process and present in supplementary materials a longer list of questions. Certainly, the wording of questions would differ in a replication of this study, and the order of the list would likely change. Furthermore, our study identified research priorities at a particular time. It is probable that over time priorities could change and new priorities emerge, especially as service provision alters. Yet, it nevertheless seems evident that our study is likely to have longevity since it has captured multiple main areas of research that are of interest across patients, carers, and professional groups. Another limitation is that our results may only represent priorities in nations with developed healthcare systems. Further research could investigate research priorities for paranoia in countries with less developed healthcare structures.

There are also methodological questions of how best to capture research priorities. The survey asked questions that many participants may not have considered previously. It is also probable that few participants had previous experience in formulating research questions, although it should be noted that telephone support was provided for participants who requested such assistance in completing surveys. Conducting semi-structured interviews within a qualitative methodological framework, or holding focus groups, could have provided greater depth and insight into participants’ priorities and the reasons for these being prioritised.^28^,^30^ Nevertheless, we believe the methods used have generated a valuable list of research questions about paranoia. Such priority-setting exercises could also prove helpful to conduct for other psychotic experiences such as hallucinations, grandiosity, and anhedonia.

## Supplementary material

10.1136/bmjment-2024-301224 online supplemental file 1

## Data Availability

Data are available on reasonable request.
