# Supplementary material for: Potential research priorities for understanding and treating severe paranoia (persecutory delusions): a priority-setting partnership between patients, carers, mental health staff, and researchers
Source: BMJ Ment Health. 2024 Dec 3;27(1):e301224. doi: 10.1136/bmjment-2024-301224 (PMC11624696; doi:10.1136/bmjment-2024-301224)
Supplement: online supplemental file 1 [file bmjment-27-1-s001.pdf]

Supplementary materials 1: The questions in the second survey

1.	**Is there cross-over between severe paranoia and social phobia and if so, what factors cause transition between the two?** *[L.Ex., Cn.]**
2.	**What premorbid characteristics or circumstances predict progression from anxiety to severe paranoia?** *[Cn., Res.]*
3.	**How does illegal drug use lead to persistent severe paranoia - including circumstances where a low number of exposures (including single dose) leads to illness as well as longer-term usage?** *[Cn., Res.]*
4.	**What are the root causes of severe paranoia?** *[L.Ex., Fam.]*
5.	**Are there differences in different ethnic groups in terms of how paranoia presents, is managed, and in recovery ?** *[Cn.]*
6.	**Is the word paranoia the most helpful way of describing the experiences involved or are there other options?** *[Cn.]*
7.	**In what ways can we promote families' understanding about what severe paranoia is?** *[L.Ex.]*
8.	**What shapes the response of families to early signs of severe paranoia?** *[Cn., Res.]*
9.	**How does severe paranoia limit the development of therapeutic relationships?** *[Cn.]*
10.	**What is the impact of severe paranoia on someone's ability to carry out everyday basic tasks (washing, cooking, buying food etc.)?** *[L.Ex.]*
11.	**What are the impacts of caring for someone with severe paranoia?** *[L.Ex.]*
12.	**What is the impact of severe paranoia on cognitive functioning?** *[Fam., Cn.]*
13.	**What can help people with severe paranoia get back to work?** *[L.Ex.]*
14.	**What is the impact on children of having parents who experience severe paranoia?** *[Cn.]*
15.	**How do people with severe paranoia manage paranoid thoughts on a day to day basis?** *[L.Ex., Fam., Cn.]*
16.	**What coping mechanisms are used for severe paranoia?** *[L.Ex.]*
17.	**How can families and carers be better equipped and supported to manage severe paranoia in loved ones?** *[L.Ex., Res.]*
18.	**As most NHS services work on a model of needing significant engagement from the person what is the best model of engagement to utilise for people with severe paranoia?** *[L.Ex., Cn.]*
19.	**How can access to services for people with severe paranoia be improved?** *[Cn.]*
20.	**What are the types of qualities in NHS or other mental health service professionals that help people with severe paranoia feel more comfortable, better able to trust, and share the difficulties that come about due to severe paranoia?** *[Cn.]*
21.	**For people who have recovered from severe paranoia, what, if anything, about NHS or other mental health services helped them, or did not?** *[Cn.]*
22.	**What does recovery mean for people diagnosed with severe paranoia?** *[Cn.]*
23.	**What is the best way to measure 'recovery' from severe paranoia?** *[Cn.]*
24.	**What are favourable and unfavourable prognostic indicators for recovery from severe paranoia (including personality and other psychological / phenomenological factors)?** *[Cn.]*
25.	**How can occupational therapy help in the treatment of severe paranoia?** *[L.Ex., Cn.]*
26.	**What should come first in treating severe paranoia: pharmacological intervention or psychological intervention?** *[Cn.]*
27.	**Which medication is most effective for severe paranoia?** *[L.Ex.]*
28.	**How can recovery from severe paranoia, especially post-therapy, be best sustained?** *[Cn., Res.]*
29.	**To what extent does forced hospitalisation and medication add to paranoid ideation?** *[L.Ex., Res.]*
30.	**Does hospital help or hinder recovery in individuals with severe paranoia?** *[L.Ex.]*
31.	**Could severe paranoia be prevented?** *[L.Ex.]*
32.	**Can peer support and soteria-informed approaches work to end severe paranoia?** *[L.Ex.]*
33.	**How can GPs and others be best supported to help people with severe paranoia in primary care?** *[L.Ex.]*
34.	**How can severe paranoia be addressed when some of the paranoia may be grounded in true lived experience, such as a violent attack resulting in constant fear of attack?** *[L.Ex., Cn.]*

**35.**	**How can the suicide risk of people who have recently recovered from severe paranoia be reduced?** *[L.Ex.]*
**36.**	**What are the percentages of people that recover from severe paranoia and to what degree do they achieve recovery?** *[Cn.]*
**37.**	**How can severe paranoia be managed outside of hospitals when it is life threatening?** *[L.Ex.]*
**38.**	**Why do some people's severe paranoia remain the same paranoid delusions during each experience of an episode (e.g. why are they always the same/similar beliefs yet always slightly more advanced each time)?** *[L.Ex.]*

**An abbreviated identifier follows each question, to indicate whether the question was suggested by: a person with lived experience of severe paranoia ['L.Ex.'], a family member of someone with lived experience ['Fam.'], a clinician ['Cn.'], or a researcher ['Res.'].*
